# Significance of galactinol and raffinose family oligosaccharide synthesis in plants

**DOI:** 10.3389/fpls.2015.00656

**Published:** 2015-08-26

**Authors:** Sonali Sengupta, Sritama Mukherjee, Papri Basak, Arun L. Majumder

**Affiliations:** Division of Plant Biology, Bose Institute, Kolkata, India

**Keywords:** raffinose synthase, stachyose synthase, galactinol synthase, stress, RFO

## Abstract

Abiotic stress induces differential expression of genes responsible for the synthesis of raffinose family of oligosaccharides (RFOs) in plants. RFOs are described as the most widespread D-galactose containing oligosaccharides in higher plants. Biosynthesis of RFOs begin with the activity of galactinol synthase (GolS; EC 2.4.1.123), a GT8 family glycosyltransferase that galactosylates *myo*-inositol to produce galactinol. Raffinose and the subsequent higher molecular weight RFOs (Stachyose, Verbascose, and Ajugose) are synthesized from sucrose by the subsequent addition of activated galactose moieties donated by Galactinol. Interestingly, GolS, the key enzyme of this pathway is functional only in the flowering plants. It is thus assumed that RFO synthesis is a specialized metabolic event in higher plants; although it is not known whether lower plant groups synthesize any galactinol or RFOs. In higher plants, several functional importance of RFOs have been reported, e.g., RFOs protect the embryo from maturation associated desiccation, are predominant transport carbohydrates in some plant families, act as signaling molecule following pathogen attack and wounding and accumulate in vegetative tissues in response to a range of abiotic stresses. However, the loss-of-function mutants reported so far fail to show any perturbation in those biological functions. The role of RFOs in biotic and abiotic stress is therefore still in debate and their specificity and related components remains to be demonstrated. The present review discusses the biology and stress-linked regulation of this less studied extension of inositol metabolic pathway.

## Introduction

Raffinose family of oligosaccharides (RFOs) are α-1, 6-galactosyl extensions of sucrose (Suc). This group of oligosaccharides is found in plants and is known to serve as desiccation protectant in seeds, as transport sugar in phloem sap and as storage sugars. The galactosyl group of RFOs is donated by galactinol (Gol; 1-*O*-α-D-galactopyranosyl-L-*myo*-inositol). Synthesis of Gol is a key and absolute requirement for entering into the pathway of RFO biosynthesis. The key enzyme galactinol synthase (GolS, EC 2.4.1.123) thus is the primary checkpoint in RFO flux, which synthesizes Gol in plants using UDP-Galactose (UDP-Gal) and L-*myo*-inositol. GolS serves as a crosslink between central inositol (Ino) metabolism and RFO biosynthesis, and also controls entry of Ino into the process.

**FIGURE 1:**
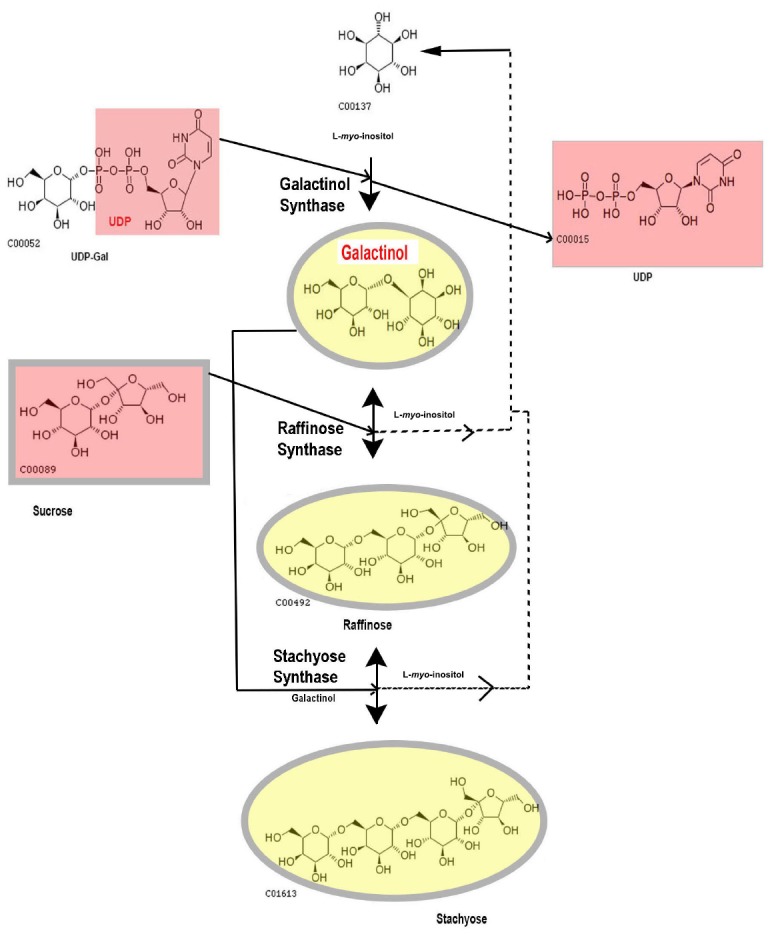
**The biochemical pathway of RFO synthesis up to Stachyose.** Compounds are shown with KEGG numbers.

Within the RFO biosynthetic pathway, the other two major enzymes are (a) raffinose synthase (RafS, EC 2.4.1.82) that transfers a galactosyl moiety from Gol to Suc and synthesizes Raffinose (Raf) (b) stachyose (Sta) synthase (StaS, EC 2.4.1.67) that further uses Gol as a galactosyl donor to Raf and produces the tetrasaccharide Sta. Both these reactions are reversible (Lehle and Tanner, 1973; Peterbauer and Richter, 1998; Peterbauer et al., 1999). There are higher molecular weight (MW) RFOs present in some plants (discussed later in this article) but Raf and Sta are the major sugars in this group and this study will concentrate on structure, function, and regulation pattern of GolS, RafS, and StaS with special reference to abiotic stress. For the biochemical pathway of RFO synthesis from Gol via Raf to Sta, refer Figure 1.

Raffinose family of oligosaccharides have a wide range of predicted functions. They are synthesized and stored in seeds protecting the embryo from maturation associated desiccation (Downie et al., 2003), participate in several cellular functions such as transport and storage of carbon, signal transduction (Stevenson et al., 2000; Xue et al., 2007), membrane trafficking (Thole and Nielsen, 2008), and mRNA export (Okada and Ye, 2009). They also act as signaling molecule following pathogen attack and wounding (Couée et al., 2006; Kim et al., 2008) and accumulate in vegetative tissues in response to a range of abiotic stresses, including freezing (Zuther et al., 2004, 2012; Hincha et al., 2005; Peters and Keller, 2009). RFOs are currently emerging as crucial molecules during stress response in plants, because of their membrane-stabilizing, antioxidant and, perhaps, predicted signaling functions (Hincha et al., 2003; Kim et al., 2008; Nishizawa et al., 2008a,b; Valluru and Van den Ende, 2011). It has also been reported that Raf exists in the chloroplast (Schneider and Keller, 2009; Foyer and Shigeoka, 2011) and may play a role in stabilizing photosystem II (Knaupp et al., 2011).

Raffinose family of oligosaccharides accumulate differentially in different plant parts. Also, plants show variability in the type of RFO they accumulate. In contrast to Raf, which is universal in occurrence, Sta and other higher degree of polymerization (DP) RFOs such as verbascose (DP5) and ajugose (DP6) accumulate in the vacuole of only certain plant species (Peters and Keller, 2009). Increased synthesis of RFOs has been reported to be linked to specific conditions like stress or storage or transport and several recent work demonstrated that GolS/RafS expression level in specific tissues is directly linked to RFO concentration in the plant (Cunningham et al., 2003; Downie et al., 2003; Volk et al., 2003).

In this article, we reviewed the phylogenetic relationship among the enzymatic isoforms of GolS/RafS/StaS in different plant species. High genetic diversity among plant kingdom regarding expression of the key enzymes in this pathway was reported and the basis of such was pondered upon.

## Reports on Synthesis and Catabolism of RFOs: The Biochemical Routes

The metabolism of RFOs has been thoroughly studied in the model plant *Arabidopsis thaliana* (Iftime et al., 2011), in *Ajuga reptans*, a member of the Lamiaceae (Peters and Keller, 2009) and in a number of legume seeds (Blöchl et al., 2005). The principal metabolites of the classical RFO pathway are the galactosylcyclitols, Gol and *myo*-inositol (Figure 1). GolS, a GT8 family glucosyltransferase galactosylates *myo*-inositol to produce Gol, as stated before. Till date, the only proven function for Gol is to serve as substrate for larger soluble oligosaccharides. The first member of this series, Raf [*O*-α-D-galactopyranosyl-(1→6)-α-D-glucopyranosyl-(1→2)-β-D-fructofuranoside] is the main RFO in most monocotyledon seeds while other RFOs, Sta, verbascose and ajugose accumulate predominantly in seeds of dicotyledons (Dey, 1985). Classic RFOs with a DP up to 15 have been found after cold treatment in *Ajuga reptans* L. (Bachmann et al., 1994). Isomers of RFOs containing α-galactosidic linkages at other carbons of the glucose (e.g., umbelliferose) or at the fructose moiety (planteose and the sesamose series) are of restricted occurrence in higher plants (Dey, 1985).

The galactosyl donors involved in RFO metabolism are UDP-D-galactose, Gol [*O*-α-D-galactopyranosyl-(1→1)-L-*myo*-inositol] and RFOs themselves. The biosynthesis of Raf is catalyzed by RafS, which is specific for Suc as a galactosyl acceptor (Lehle and Tanner, 1973). Raf is used subsequently for the synthesis of Sta by the enzyme StaS (Tanner and Kandler, 1968). In addition to this well-established biosynthetic route, higher DP RFOs may be produced by the action of a Gol-independent enzyme, called galactan:galactan galactosyltransferases (GGTs; Bachmann et al., 1994). It has been reported that galactosylcyclitols (galactosyl ononitol and galactopinitol A) may as well serve as galactosyl donors in the biosynthesis of Sta (Richter et al., 2000). In buckwheat (*Fagopyrum esculentum*), a multifunctional GolS was found that shares homology with fagopyritol synthase (FeGolS). Two *FeGolS* genes were characterized that were involved in the synthesis of fagopyritols (galactosylcyclitols) utilizing UDP-galactose and D-*chiro*-inositol (Ueda et al., 2005).

Raffinose family of oligosaccharides catabolism in plants has received relatively little attention but is equally important compared to the synthesis reaction. They are digested by acid and alkaline α-galactosidases sequentially so as to remove the terminal Galactose residues (Madore, 1995; Zhou et al., 2012a,b). Sucrose may be digested to fructose and glucose by invertase or to fructose and UDP-Glucose by Suc synthase. Fructose, glucose and UDP-Glucose can then readily enter other metabolic pathways. Some seed imbibition proteins are actual homologs of the alkaline α-galactosidases and along with invertases they may be involved in the degradation of RFOs (Van den Ende, 2013). Further importance of the reverse reactions catalyzed by RafS/StaS in the maintainance of RFO flux are discussed in later sections.

## Physiological Importance of RFO Synthesis

The physiological importance and absolute requirement of RFO in a system remain elusive even after decades of study. RFO biosynthetic pathway is essentially an extension of inositol metabolic pathway (Loewus and Murthy, 2000; Sengupta et al., 2012). Unlike inositol-conjugated lipids, methylated inositols or cell-wall polysaccharides, which are other conjugation pathways in which inositol is known to participate; RFOs do not appear to be functionally directly related to stress amelioration in plants under natural condition. Truly enough, RFOs have the beneficial properties of a compatible solute. *In vitro* studies claim that RFOs insert themselves within the lipid headgroups of membrane bilayer and stabilizes it when disrupting conditions prevail (Hincha et al., 2003). Their high oligomeric length may serve to protect liposomes (Cacela and Hincha, 2006) and probably also act as a free radical scavenger (Nishizawa et al., 2008b). The other major evidence supporting that RFOs have potential roles in stress amelioration is the accumulation of RFOs, especially Raf, under stresses like heat, cold, salinity and drought (Santarius and Milde, 1977; Bachmann et al., 1994; Taji et al., 2002; Pennycooke et al., 2003; Panikulangara et al., 2004; Nishizawa et al., 2008a; Peters and Keller, 2009; Peters et al., 2010). From these experiments, it seems that RFOs may be important in stress protection; however, contradictory evidences also indicate that unlike an essential stress-ameliorator, genetic elimination of these biosynthetic enzymes are not lethal or highly detrimental for a plant (Panikulangara et al., 2004).

The expression of the key enzyme *GolS* is known to be linked to both abiotic stress and developmental stages. Most of the plant species reported so far exhibited multiple isoforms of *GolS*. Seven *GolS* isoforms were identified in *Arabidopsis* of which *AtGolS1* and *AtGolS2* were induced by drought, salt, or heat stress, whereas *AtGolS3* was cold induced (Taji et al., 2002). As a transgene, their over-expression resulted in isolated or combined accumulation of Gol, Raf and Sta and subsequently, enhanced tolerance to drought, salinity or cold stress (Taji et al., 2002; Panikulangara et al., 2004). Expression of *Arabidopsis GolS1* and *GolS2* was found to be regulated by a heat shock transcription factor (HSF; Panikulangara et al., 2004; Busch et al., 2005; Schramm et al., 2006). The transcription of *GolS1* and *GolS2* were induced by a combination of high light and heat stress or treatment with hydrogen peroxide in *Arabidopsis* (Nishizawa et al., 2008a). Three other putative *GolS* (*GolS8*–*GolS10*) genes were identified from *Arabidopsis* genome database (Nishizawa et al., 2008a) but their regulation patterns are not studied in detail.

*AtGolS1* mutant plants fail to accumulate heat stress-induced Gol and Raf (Panikulangara et al., 2004), suggesting that *AtGolS1* may be the principle *GolS* isoform responsible for heat stress induced Raf or Gol accumulation. It is also reported that such failure does not prove detrimental to the plant. However, Peters et al. (2010) generated a double mutant (*GolS1* T-DNA insertion mutant in *GolS2* background) and claimed that despite a clear transcriptional elevation of *GolS1* in *GolS2* mutants, they remain hypersensitive to water stress, exhibit rapid loss of water and lower enzymatic activity. The double mutants are clearly drought-hypersensitive. Although *Arabidopsis* neither stores nor transports RFO, such observations suggest that the biosynthetic pathways that are supplied to by different *GolS* isoforms may contribute to stress protection.

In the study of RFO physiology, the genetic tools that have been exploited heavily are overexpression (OE) or knockout (KO) lines. OE of a *Medicago falcata GolS* (*MfGolS1*) in tobacco resulted in elevated tolerance to freezing and chilling in transgenic plants along with enhanced levels of RFOs. Osmotic resistance was reportedly increased in the transgenic tobacco plants (Zhuo et al., 2013). Another *GolS* gene that was dehydration and ABA-inducible in the resurrection plant *Boea hygrometrica* conferred high dehydration tolerance in a transgenic tobacco system (Wang et al., 2009).

Two major problems are associated with the OE approach. Firstly, as shown by Taji et al. (2002) constitutive OE of *GolS2* hyperaccumulates Gol and subsequently imparts stress tolerance. However, all compatible solutes have been reported to impart osmotolerance upon hyperaccumulation (Tarczynski et al., 1992; Thomas et al., 1995; Holmstrom et al., 1996, 2000; Alia et al., 1998; Nanjo et al., 1999; Hong et al., 2000; Huang et al., 2000; Han et al., 2005). OE of a compatible solute in cell does not necessarily represent a natural physiological event. Secondly, RFO catabolism may exert significant effects on accumulation status of these sugars. Madore (1995) proposed that Sta (when present) may be hydrolyzed by an alkaline alpha-galactosidase and produce Raf. This may increase the concentration of Raf in the cell unless it is reverse–hydrolyzed by RafS and the resulting Gol is reverse hydrolyzed to *myo*-inositol and UDP-Gal (Gao and Schaffer, 1999). These forward and reverse reaction possibilities make it hard to draw a direct correlation between RFO accumulation and physiological stress protection. Reverse genetic tools can be of better assistance, however sufficient knowledge of the functional areas of RFOs are necessary. The physiological interpretation of isoform-specific stress resistance may be flawed since constitutive OE of a conjugation pathway carries the possibility of draining too much inositol, which is diversified in many life processes and such processes are also known to impart the basic stress tolerance in a plant.

## Location Specificity of Expression

The location of RFO synthesis and/or GolS/RafS/StaS expression in plant therefore emerges as an immensely important control of RFO biosynthesis. All three genes exhibit more than one isoforms with differential expression in specific tissue location or in response to stress. For example, in *Ajuga reptans* two distinct cold inducible *GolS* genes are transcribed in discrete locations (*GolS1* in mesophyll cells and *GolS2* in companion cells of phloem; Sprenger and Keller, 2000). Moreover, in *Ajuga reptans*, two RFO pools are present, one is the long-term RFO storage pool in mesophyll and the other is the RFO transport pool (Bachmann et al., 1994). Sprenger and Keller (2000) showed that *GolS1* is primarily responsible for synthesis of RFO storage pool in *Ajuga* whereas *GolS2* synthesizes the Gol that enters into the transport RFO pool. The functional separation is achieved by means of localized expression of the genes and anatomical barrier between the metabolites (Sprenger and Keller, 2000). It is also important to note that it is not known which of these two pools take part in amelioration of stress.

Various reports highlight tissue specific expression of *GolS*. A seed-specific *GolS* from *Lycopersicum esculentum* (*LeGolS-1*) confer desiccation tolerance to the seeds (Downie et al., 2003). RNAi suppression of two *GolS* isoforms expressed in the intermediary cells of *Verbascum phoeniceum* inhibits RFO synthesis (McCaskill and Turgeon, 2007). Three *GolS* isoforms isolated from *Zea mays* (*ZmGOLS*, *2* and *3*) found to be associated with abscission of developing seeds and callus (Zhao et al., 2004a,b,c). A *GolS* gene reported from *Coptis japonica* (*CjGolS*) is involved in berberine tolerance (Takanashi et al., 2008). Study of three *GolS* genes from *Coffea arabica* evaluated their differential regulation under several abiotic stresses (water deficit, high salt, and heat stresses). The three *CaGolS* were highly expressed in leaves with little to no expression in “flower buds, flowers, plagiotropic shoots, roots, endosperm and pericarp of mature fruits” (Santos et al., 2011). Three hybrid poplar *GolS* homologs showed spatial and temporal expression pattern both diurnally and annually (Unda et al., 2011). Zhou et al. (2012a,b) cloned a *GolS* from cotton (*GhGolS*) that showed tissue specific expression pattern in leaves, anthers and fibers and is localized to cell membrane.

Raffinose synthase (*RafS*) is highly specific for Gol and Suc, acting as donor and acceptor respectively. *RafS* also shows multiple isoforms in higher plants but their specificity is underestimated. *RafS* or *RafS*-like sequences were reported from lower to higher group of plants, e.g., *Chlamydomonas reinhardtii*, *Physcomitrella patens*, *Glycine max* (Oosumi et al., 1998), *Vicia faba* (Watanabe and Oeda, 1998), and *Pisum sativum* (Peterbauer et al., 2002). Six putative *RafS* genes (*RS1*-*RS6*) were identified from the *Arabidopsis* genome databases, two of which were overexpressed in tobacco and showed tolerance to oxidative stresses (Nishizawa et al., 2008a). In rice, 10 *RafS* or *RafS*-like sequences have been identified (Saito and Yoshida, 2011). Little is known about the structural aspects of RafS, although there is a growing body of interest in exploiting the enzyme commercially (Oosumi et al., 1998; Watanabe and Oeda, 1998; Peterbauer and Richter, 2001). Reports of *StaS* structure are further sparse as they bear sequence resemblance to *RafS*. *StaS* has been purified from seeds of kidney bean, adzuki bean and lentil (*Lens culinaris*, Tanner and Kandler, 1968; Peterbauer and Richter, 1998; Hoch et al., 1999). The association of Sta biosynthesis with minor veins was confirmed in *Cucumis melo* through immunolocalization (Holthaus and Schmitz, 1991). Unlike *RafS*, *StaS* shows a broad substrate specificity that includes galactosylcyclitols (Gol, galactosyl ononitol and galactopinitol A) with *myo-*inositol and methylated inositols (ononitol and pinitol), as acceptors (Peterbauer and Richter, 1998, 2001; Hoch et al., 1999). A multifunctional *StaS* catalyzing synthesis of Sta and verbascose had been characterized from developing pea (*Pisum sativum*) seeds (Peterbauer et al., 2002). Gol dependent enzyme activities for higher RFOs were partially purified from seeds of pea (*Pisum sativum*), *Vicia faba* and *Vicia sativa* (Tanner et al., 1967; Kandler and Hopf, 1980). Thus it seems that the RFO biosynthetic genes are either expressed in seeds or in phloem tissue. *GolS*, *RafS*, and *StaS* are believed to be localized in the cytosol, although, in the leaves the RFOs (metabolites) might enter the vacuole and the chloroplasts (Nägele and Heyer, 2013) and sometimes may be stored in mesophyll cells. It is not understood which of these pools may participate in stress amelioration.

## The Evolutionary Aspect

**FIGURE 2:**
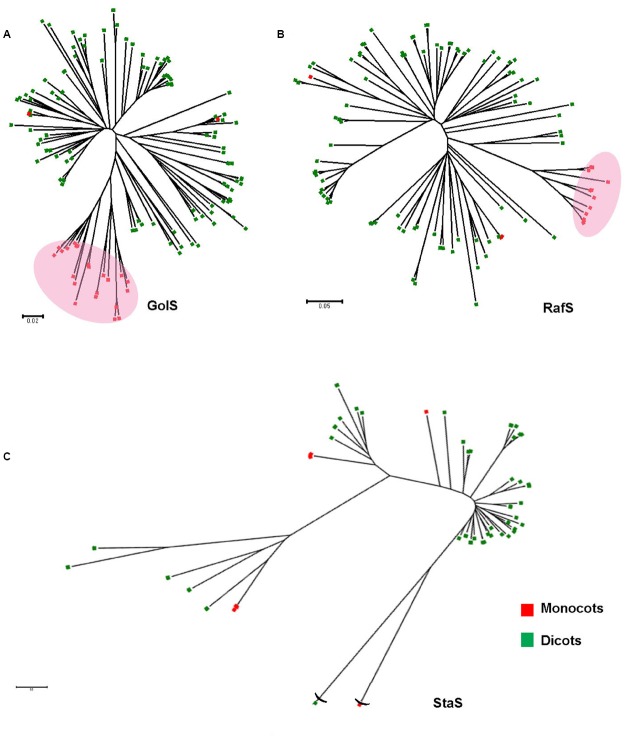
**The evolutionary relationships of taxa representing diversification of reported GolS (A), RafS (B), and StaS (C) protein sequences within the plant kingdom.** The red blocks and green blocks represent monocot and dicot species, respectively. The evolutionary history was inferred using the Neighbor-Joining method. The optimal tree with the sum of variable branch length = 20.18266976 is shown. The tree is drawn to scale and bootstrap test was performed with 500 replicates, with branch lengths in the same units as those of the evolutionary distances used to infer the phylogenetic tree. The evolutionary distances were computed using the Poisson correction method and are in the units of the number of amino acid substitutions per site. The rate variation among sites was modeled with a gamma distribution (shape parameter = 1). The analysis involved 67/150/45 amino acid sequences. All ambiguous positions were removed for each sequence pair. Evolutionary analyses were conducted in MEGA6 (Zuckerkandl and Pauling, 1965; Felsenstein, 1985; Saitou and Nei, 1987; Tamura et al., 2013).

The notable feature of RFO biosynthesis is that this pathway is restricted to higher plants. We have shown earlier (Sengupta et al., 2012) that GolS is a member of GT-8 group of galactosyltransferases, and among the other GT-8 enzymes it has a special pattern of evolution. In a comprehensive accumulation of GolS and GolS-like sequences among plant kingdom, it was evident that although closely related to the other group of GT-8, GolS are special in evolutionary and structural perspective (Sengupta et al., 2012). The phylogenetic configuration of RafS among plant kingdom correlates to GolS (Figure 2). The monocots are grouped into a small clade from the base of which dicots diverge and many distinct families form well defined clusters. A similar pattern of family diversification is observed between GolS and RafS, but StaS is disarrayed and do not correlate well (Figure 2). Although this study is primarily suggestive and preliminary, similar diversification pattern of GolS and RafS probably indicates similar evolution pattern and timeframe whereas StaS (or higher DP RFOs) may not have co-evolved with them. A need of further and deeper analysis of the evolutionary patterns are indicated which may split the RFO biosynthetic scheme in two parts: the initial synthesis up to Raf and the later part for synthesis of higher DP RFOs.

## Stress Specificity of RafS/GolS Expression

There is a plethora of literature dedicated to stress-linked accumulation of RFOs. All plants, at some point, synthesize some RFOs, but many of them like rice or *Arabidopsis* neither transport nor accumulate large quantities in their tissues and/or organs (Amiard et al., 2003). RFOs undoubtedly accumulate in many plant groups in response to diverse abiotic stresses (Nishizawa et al., 2008a,b) such as temperature extremes or drought. Under extreme conditions, RFOs are thought to act as osmolytes to maintain cellular integrity and function (Nishizawa et al., 2008a,b) if they can reach the required level of accumulation. In ABA-deficient and ABA-insensitive double-mutant seeds of *Arabidopsis* (*aba-1*, *abi3-1*), which are viable but desiccation-intolerant, ABA treatment *in vitro* increased seed Raf content and promoted desiccation tolerance (Ooms et al., 1994). This proposes an involvement of ABA in regulation of RFO metabolism. ABA suppresses germination processes, which are usually associated with RFO degradation, and thus, altered RFO levels in ABA-deficient or ABA-insensitive mutants might well be the consequence of germination associated catabolism (Brenac et al., 1997). RFOs have significant report of accumulation in low temperature stress. *Ajuga reptans* (Peters and Keller, 2009), alfalfa (Cunningham et al., 2003), *Arabidopsis* (Klotke et al., 2004), cabbage (Santarius and Milde, 1977), salt grasses (Shahba et al., 2003), spruce (Wiemken and Ineichen, 1993), petunia (Pennycooke et al., 2003) as well as the photoautotrophic alga *Chlorella vulgaris* (Salerno and Pontis, 1989) show cold-induced RFO accumulation.

Reports of accumulation of Raf in the unicellular organisms are not obtained. So, the role of Raf in a unicellular organism is unknown. *SIP* genes/*RafS* genes are a phylogenic relative of alkaline α-galactosidase genes, which are responsible for degradation of galactosidic bonds. Moreover, *AtSIP2* (At3G57520) is a Raf-specific AGA (Peters et al., 2010) Thus, SIP proteins, although alternatively referred to as RafS proteins, and occurring in lower plants may not always produce Raf.

## The Possible Signaling Role

There are reports of Gol switching on early pathogen-attack related transcripts (such as *PR1a*, *PR1b*, and *NtACS1*) in tobacco (Kim et al., 2008), suggesting a role in biotic stress signaling. Gol induces the expression of the *PR-1a* gene, via a salicylic acid-dependent pathway (Couée et al., 2006). Both *GolS* and *RafS* contain W-box *cis*-elements in their promoters, regulated by ABA-inducible WRKY (Wang et al., 2009). This suggests a possible role of *GolS* and *RafS* downstream ABA signaling. A recent study demonstrated that starch hydrolysis results in hexose and Raf accumulation during the first 24 h after a cold shock treatment in *Arabidopsis*. The importance of such accumulation remains unknown but it has been suggested that Raf may take part in chilling-induced ROS homeostasis (Sicher, 2011).

Nishizawa et al. (2008a,b) reported tolerance to oxidative stress in *GolS* and *RafS* overexpressing transgenic plants predicting a role of Gol and Raf as scavengers of ROS, thus playing a novel role in the protection of cellular metabolism. Presence of a Raf transporter (raf) in chloroplast membrane has been established, although whether they help in maintaining chloroplast membrane integrity under oxidative stress, is not known (Schneider and Keller, 2009; Valluru and Van den Ende, 2011).

## Storage and Transport Roles of RFOs

Some plants may store RFOs in large concentrations, sometimes 25–80% of their dry weight, in tubers (French, 1954; Keller and Matile, 1985) and in photosynthesizing leaves where they are localized in mesophyll cells (Senser and Kandler, 1967; Bachmann et al., 1994). RFOs predominantly accumulate in seeds (Korytnyk and Metzler, 1962; Handley et al., 1983a,b; Blackman et al., 1992; Haritatos et al., 2000; Peterbauer et al., 2002). Haritatos et al. (2000) found little accumulation in the leaves. The molecular handles of such differential accumulation are not clearly known; however, RFOs accumulate late in seed development, starting at about the beginning of seed fill and continuing up to maturation drying. They are deposited in all parts of the seed (endosperm, embryo and the seed coat), although the levels of individual α-galactosides may vary considerably in these tissues (Kuo et al., 1988; Horbowicz and Obendorf, 1994; Frias et al., 1999). Reduction of the content of *myo*-inositol in tubers of transgenic potato (*Solanum tuberosum* L.) resulted in strongly reduced levels of Gol and Raf (Keller et al., 1998). GolS is also purified from the cotyledons of kidney bean (*Phaseolus vulgaris*, Liu et al., 1995) and mature zucchini leaves (*Cucurbita pepo*, Smith et al., 1991). RFOs protect the embryo during the desiccation that occurs during seed maturation and thus play important role in prolonged seed survival (Peterbauer et al., 2002).

Raf and Sta also serve as the main transportable solute in the orders Lamiales, Cucurbitales, Cornales, and in one family of the Celastrales and are mechanistically linked with phloem loading (Zimmermann and Ziegler, 1975; Haritatos et al., 1996; Hoffmann-Thoma et al., 1996; Turgeon et al., 2001). The structural and anatomical specificities of plants that drive accumulation of RFOs as major transport sugar are reviewed elsewhere (Lemoine et al., 2013). The phloem loading function of RFOs is best studied in *Ajuga reptans* (common bugle). In this frost tolerant evergreen Labiatae, (i) Sta is the main carbon translocate; (ii) higher RFO oligomers are the main carbon store (Bachmann et al., 1994); and (iii) higher RFO oligomers are synthesized by GGT which is targeted to the vacuole via a novel sorting determinant (Bachmann et al., 1994; Haab and Keller, 2002; Tapernoux-Lüthi et al., 2004, 2007). There are two RFO pools in its leaves: a storage pool associated with leaf mesophyll and a transport pool associated with the phloem-loading sites (Bachmann et al., 1994) where Raf and especially Sta are produced and loaded in the phloem, according to the polymer trapping model (Turgeon, 1991). These two pools rely on different *GolS* isoforms (Sprenger and Keller, 2000). Haritatos et al. (2000) cloned a *GolS* gene from melon (*Cucumis melo*) and studied the expression pattern in *Arabidopsis* and cultivated tobacco. The expression pattern is consistent with the loading function, i.e., gene expression is limited to the minor vein network.

From the classic study of Rennie and Turgeon (2009) a species-specific pattern of transport sugars can be drawn. The anatomy of phloem remains highly important in this pattern, especially the occurrence of intermediate companion cells (Gunning and Pate, 1969; Turgeon et al., 1975). Ordinary companion cells (OC), transfer cells (TC), and intermediate cell (IC) are the three types of companion cells found in phloem. Transfer cells have hemicellulose ingrowths and no plasmodesmatal connection with the mesophyll cells; ordinary companion cells have one plasmodesmatal connection with the mesophyll cells and ICs, first discovered in minor veins of cucurbits (Turgeon et al., 1975) show a specialized smooth ER and abundant secondary branched plasmodesmata connected to sieve cells and bundle sheaths (Turgeon and Webb, 1976; Zambryski and Crawford, 2000). RFOs are loaded into phloem suggestively in symplastic type II plants using polymer trapping model. Briefly, Suc from source cells (mesophyll) moves into the ICs via bundle sheath where the enzymes for RFO biosynthesis are localized. The RFOs (Raf/Sta) cannot diffuse back to the source because of their higher size, and that traps them in the ICs. The only way to move is within the sieve cells and due to the high osmotic pressure built up, the sugars are thus loaded into the sieve cells. This model is highly species specific, and most of the experimental evidences come from the Cucurbitaceae.

## Conclusion

The two predominant roles of RFOs in plants are transport and storage. Stress increases the expression of the genes in this pathway. Presumably all RFOs, including higher oligomers, may exert protective effects when accumulated in a higher concentration in a cell that expand their functional significance (as phloem and storage carbohydrates) to include stress protection (Peters and Keller, 2009) but it is still not an exclusive role for RFOs.

In this review, we evaluated and described the literature available on GolS/RafS/StaS gene expression and protein synthesis as well as RFO accumulation in response to stress. We have preliminarily assessed the evolution pattern of the three major genes and concluded that although RafS and GolS follow somewhat similar evolutionary pattern within the plant kingdom, StaS, despite its high sequence similarity with RafS, follows a somewhat different pattern. We also observed that there are multiple RafS/RafS-like proteins in higher plants that may or may not be functional and may also have very different physiological roles. Such different roles have not been studied very well. In spite of the stress-associated upregulation in many plants, the significance of RFO biosynthetic pathway across plant kingdom has not been clearly elucidated. OE of RFO synthesizing genes and increase in RFO levels improve stress tolerance in the plant, but loss of the gene does not compromise the normal physiology. Recently, Cao et al. (2013) expressed RafS/StaS/GolS directly into the ordinary companion cells of *Arabidopsis* to introduce RFO in the main translocation stream. Not only they observed an altered translocation stream, but they also reported lower fecundity of aphid feeding, which is not due to direct toxicity of RFO but the choice of aphids to feed on Suc-translocating plants (Cao et al., 2013). This observation may have broader perspectives from the evolutionary or ecological viewpoint. To conclude, evolution of the molecular physiology of RFO biosynthesis is still an interesting question, and more insights into the reactions, molecules and significance are needed to resolve it.

### Conflict of Interest Statement

The authors declare that the research was conducted in the absence of any commercial or financial relationships that could be construed as a potential conflict of interest.

## Abbreviations

DP: degree of polymerization
Gol: galactinol
GolS: galactinol synthase
MW: molecular weight
Raf: raffinose
RafS: raffinose synthase
RFO: raffinose family of oligosaccharides
Sta: stachyose
StaS: stachyose synthase.
